# Construction of Durable Self-Cleaning PDMS Film on Polyester Fabric Surface

**DOI:** 10.3390/ma16010052

**Published:** 2022-12-21

**Authors:** Yong Xia, Nan Zhu, Ying Zhao, Jiehui Zhu, Huajie Chen, Liyun Xu, Lirong Yao

**Affiliations:** 1 National & Local Joint Engineering Research Center of Technical Fiber Composites for Safety and Protection, Nantong University, Nantong 226019, China; 2 College of Textile and Clothing, Nantong University, Nantong 226019, China

**Keywords:** self-cleaning polyester, polydimethylsiloxane, waterborne polyurethane, atmospheric pressure plasma

## Abstract

The superhydrophobic surface can be prepared by two methods; one is by reducing the surface energy, and the other is by constructing a micro-nano rough structure. To achieve high superhydrophobic performance in terms of durability, the firm combination of hydrophobic coating and substrate is particularly important. Here, we use polydimethylsiloxane (PDMS) as a low surface energy monomer, water-borne polyurethane (WPU) as a dispersing aid, and use high-power ultrasound to disperse PDMS in water to make emulsion. The polyester matrix is etched by atmospheric plasma, dipped in PDMS emulsion, dried, and finally baked to induce PDMS on the surface of polyester fiber to cross-link into film. A series of tests on the self-cleaning polyester fabric prepared by this method show that when the concentration of PDMS is 8 g/L and the mass ratio of PDMS to WPU is 20:1, the water contact angle (WCA) reaches the maximum value of 148.2°, which decreases to 141.5° after 200 times of washing and 138.6° after 5000 times of rubbing. Before and after PDMS coating, the tensile strength of polyester fabric increases from 489.4 N to 536.4 N, and the water vapor transmission decreases from 13,535.7 g/(m^2^·d) to 12,224.3 g/(m^2^·d). This research is helpful to the large-scale production of self-cleaning polyester fabric. In the future, on the basis of this research, we will add functional powder to endow self-cleaning polyester fabric with higher hydrophobicity and other properties.

## 1. Introduction

With the rapid development of society, the textile function requirements of people are increasing, and the number of people who prefer superhydrophobic textiles is also increasing [1,2,3]. Superhydrophobic textiles have a wide range of applications [4,5], including medical, outdoor, and military applications [6,7,8]. The mechanism of the superhydrophobic phenomenon was revealed by German botanists Barthlott and Neinhuis in 1997 [9,10]. The superhydrophobic surface of plants represented by lotus leaves was constructed using a foliar microstructure and hydrophobic waxy substances, clarifying the relationship between the superhydrophobic surface and self-cleaning [11,12,13]. The main hydrophobic groups of lotus leaf fat wax are C-H and C-O, and the papilla structure with waxy coating constitutes the rough surface of leaves [14,15,16]. Years of research has shown that the methods of constructing superhydrophobic surfaces are changing the chemical composition of the surface to reduce the surface energy and constructing micro-nano rough structures on the surface [17,18,19].

Luo et al. [20]. prepared a polyurethane/silica composite superhydrophobic coating with a good wear resistance using the two-step sol-gel method. After the wear resistance test, the coating maintained good hydrophobicity and high transparency under visible light, and the maximum contact angle of the coating could reach 162.1°. Gong et al. [21]. prepared a new type of superhydrophobic coating by spraying F-SiO_2_ nanoparticles, epoxy resin adhesive, fluorosilicone varnish, and white fluorinated polyurethane coating. After 20 continuous wear cycles, 1.5 h of dripping or 20 tape peeling cycles, the contact angle of the superhydrophobic coating exceeded 150°. Phuong Nguyen-Tri et al. [22]. used alkali and plasma etching treatment, added silica nanoparticles and tetraethoxysilane, and prepared a superhydrophobic cotton fabric with a contact angle up to 173° using different input variables and etching processes. Lin et al. [23]. dipped the cotton fabric activated by O_2_ plasma in an ethanol suspension containing tetraethoxysilane (TEOS), hydroxyl-terminated polydimethylsiloxane (HPDMS), and ammonium polyphosphate (APP) and then added ammonia water after stirring, which initiated the in situ sol-gel reaction between the TEOS and the HPDMS to generate a PDMS-silica hybrid material (PDMS-silica). The micro-nano structure was prepared on the cotton fabric by the PDMS-silica and the APP.

The problems of a complicated preparation process, environmental pollution and difficulty in realizing large-scale production in the preparation process of super-hydrophobic fabrics at present must be considered. In this study, polydimethylsiloxane (PDMS) was used as a low surface energy monomer, water-based polyurethane (WPU) was used as a dispersing agent, and water was used as a dispersing medium. A durable PDMS hydrophobic coating was constructed on the surface of polyester fiber by a simple process of plasma etching at atmospheric pressure, dipping PDMS emulsion and inducing PDMS crosslinking at a high temperature. Fluorine-containing substances and organic solvents are not used in the whole preparation process, and the plasma used is atmospheric pressure plasma, which is expected to realize large-scale production [20,24,25]. Our goal is to realize the scale and pollution-free production of self-cleaning polyester fabric.

## 2. Materials and Methods

### 2.1. Materials

Knitted polyester (Zhejiang Miandu Textile Co., Ltd. Yiwu, China), polydimethylsiloxane (PDMS, molecular weight: 230 g/mol, Shenzhen Shijun Technology Co., Ltd., Shenzhen, China), waterborne polyurethane (WPU, solid content 32%, Shenzhen Jitian Chemical Co., Ltd., Shenzhen, China), and detergent 209 (Guangzhou Wangnilai Chemical Co., Ltd., Guangzhou, China).

### 2.2. Pretreatment of Polyester Fabric

A 2 g/L detergent 209 solution was prepared. Then, an appropriate amount of Na_2_CO_3_ was added to adjust the pH to 8–9, and the fabric was soaked in it (bath ratio 50:1). Subsequently, the fabric was ultrasonically cleaned at 30 °C and 40 kHz for 40 min and then washed with deionized water 3–5 times until no detergent residue remained. Finally, the fabric was dried in a 60 °C oven and then taken out for later use [26,27].

### 2.3. Preparation and Characterization of PDMS Emulsion

Weigh 1 g, 2 g, 3 g, 4 g, and 5 g PDMS in turn into a beaker filled with 500 mL deionized water to form a group of samples. Repeat the above operation to obtain the same three groups of samples [28]. Continue to add WPU into the beaker, and control the mass ratio of PDMS to WPU in the three groups of samples to be 30:1, 20:1, and 10:1, respectively. At last, three groups of samples were ultrasonically dispersed at 600 W and 40 kHz for 3 h, and three groups of emulsions with PDMS concentrations of 2 g/L, 4 g/L, 6 g/L, 8 g/L and 10 g/L were prepared [29].

The particle size of PDMS emulsion was characterized by particle size analyzer, the morphology of PDMS emulsion was observed by optical microscope, and the stability of PDMS emulsion was characterized by Zeta potential tester.

### 2.4. Preparation and Characterization of Self-Cleaning Polyester Fabric

The polyester fabric was treated using a plasma surface processor at normal temperature and pressure. The treated polyester fabric was immersed in the PDMS emulsion, and ultrasonic treatment was carried out at 40 kHz for 10 min for the PDMS to uniformly attach to the polyester fabric [30,31]. The sample was taken out with a glass rod and dried in an oven at 60 °C for 30 min. Finally, the fabric was baked at 130 °C for 10 min, and the PDMS on its surface was induced to cross link into a film [32,33].

Before and after treatment, scanning electron microscopy (SEM) was used to observe the polyester fabric, and a three-dimensional profilometer was used to characterize the surface roughness. Energy Dispersive Spectroscopy (EDS) was used to analyze the elements, X-ray photoelectron spectroscopy (XPS) was used to analyze the chemical elements and functional groups, and differential scanning calorimeter (DSC) was used to analyze the thermal properties of the self-cleaning polyester fabric.

### 2.5. Water Repellency Test

An OCA15EC contact angle measuring instrument was used to measure the water contact angle of the self-cleaning polyester fabrics with different washing times. The droplet amount was 5 μL, and the photo taking time was 60 s. Each group of samples was tested 5 times, and the average value was taken.

### 2.6. Washing Fastness Test

According to the soaping fastness method (GB/T 3921-2008, China), a SW-12JG washing fastness tester was used to test the washing fastness of the self-cleaning polyester fabric.

### 2.7. Test of Rubbing Fastness

According to the color fastness to rubbing method (GB/T 3920-2008, China), a ZJ-339-GSR color fastness to rubbing tester was used to test the color fastness to rubbing of the self-cleaning polyester fabric.

### 2.8. Spray Wetting Grade Test

According to ISO 4920-2012, that is, “Determination of Moisture Resistance of Textile Surface (Spray Test)”, the moisture resistance of self-cleaning polyester fabric with different washing times was measured.

### 2.9. Oil–Water Separation Efficiency Test

The self-cleaning polyester fabric was placed at the filter outlet of the solvent filter and clamped. Dichloromethane was selected as the heavy oil, 50 mL of which was mixed with 50 mL of dye water with methyl blue dye and then poured into a filter cup above the solvent filter, starting the separation. After completing the separation, the volume of the remaining water above *X* mL was measured, and the filtration efficiency was calculated according to the following formula:

(1) $$\mathrm{Filtration}\;\mathrm{efficiency}=\frac{X}{50}\;\times \;100\%.$$

## 3. Results and Discussion

### 3.1. Preparation and Characterization of PDMS Emulsion

**Figure 1:**
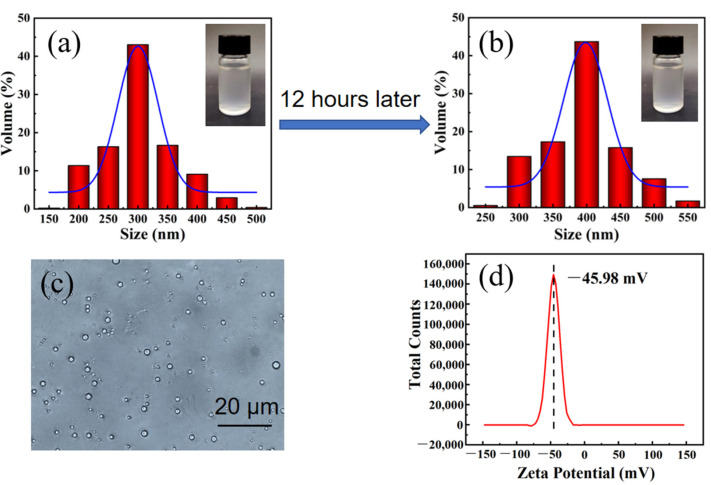
(**a**) particle size distribution of PDMS emulsion; (**b**) particle size distribution of PDMS emulsion after storage for 12 h; (**c**) optical microscope of PDMS emulsion; and (**d**) Zeta potential of PDMS emulsion.

PDMS is insoluble in water, and PDMS emulsion was prepared by dispersing it in water with high-power ultrasound. As PDMS has not been hydrophilically modified, its dispersion stability is poor [28]. When PDMS is added, a certain amount of WPU can improve the dispersion stability of PDMS emulsion. The reason is that WPU contains both hydrophilic groups and hydrophobic groups, and plays the role of surfactant. The prepared PDMS emulsion is translucent, and its particle size is about 300 nm (Figure 1a). After 12 h, the emulsion does not appear to have obvious stratification, and the particle size becomes about 400 nm (Figure 1b), which proves that the PDMS emulsion can be stably dispersed for one day. It can also be seen from Figure 1c that the particle size of PDMS emulsion is relatively uniform, and the Zeta potential of PDMS emulsion is −45.98 mV (Figure 1d). Usually, when the absolute value of Zeta potential is in the range of 40–60, it indicates that the dispersion system is stable. Therefore, we think that it is an economical, environmentally friendly and efficient method to make PDMS uniformly dispersed in water with the help of WPU.

### 3.2. Preparation and Characterization of Self-Cleaning Polyester Fabric

**Figure 2:**
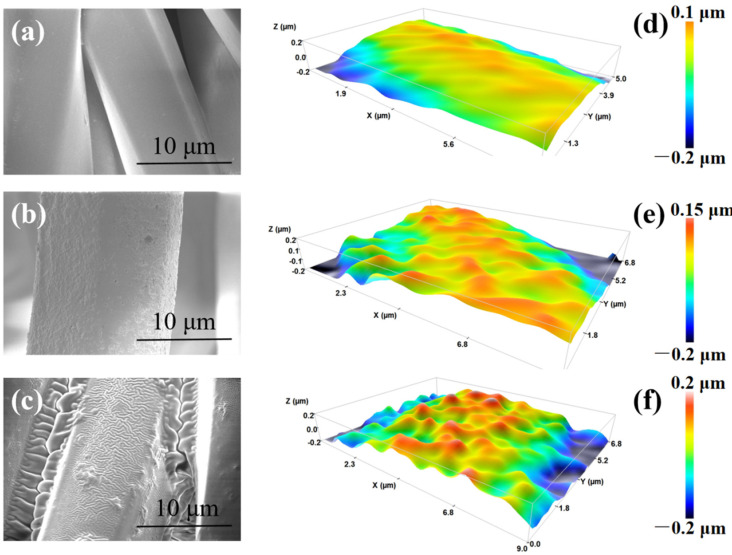
SEM shows (**a**) polyester fiber as it is; (**b**) polyester fiber after plasma etching; (**c**) polyester fiber after PDMS coating; and three-dimensional outline shows (**d**) polyester fiber as it is; (**e**) polyester fiber after plasma etching; and (**f**) polyester fiber after PDMS coating.

As shown in Figure 2a,d, the untreated polyester fiber surface is smooth and flat, while the plasma-treated polyester fiber surface has dense and uniform grooves (Figure 2b,e). The reason is that, under the bombardment of plasma energy, some C-C, C-O and C=O in the polyester macromolecular chain are broken, and small molecules are constantly bombarded, which forms an etching effect on the original smooth polyester fiber surface and increases the surface roughness and specific surface area of polyester fiber. In addition, the hydrophilic modification of polyester fiber can be carried out briefly by plasma treatment at normal pressure, which makes the original hydrophobic polyester fabric have better wettability when immersed in PDMS emulsion. The plasma-treated polyester fabric is immersed in PDMS emulsion. Under the ultrasonic condition, PDMS macromolecules will enter the grooves on the surface of the polyester fiber and uniformly adhere to the rough surface of the polyester fiber. After being taken out and dried, the PDMS molecules on the surface of the polyester fiber will cross-link at a high temperature of 130 °C, and the surface of the polyester fiber will be covered with a PDMS film. As the surface of the etched polyester fiber has a certain roughness, the film on its surface also forms a certain wrinkle structure (Figure 2c,f) and can be firmly combined with the substrate.

**Figure 3:**
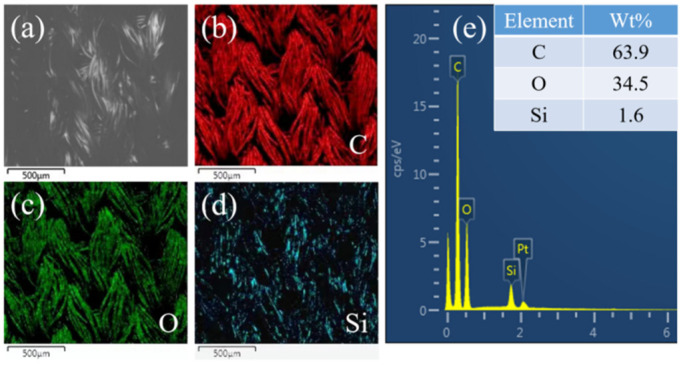
EDS mapping diagram of PDMS coated polyester fabric. (**a**) original appearance, (**b**) C element, (**c**) O element, (**d**) Si element, (**e**) element distribution.

**Figure 4:**
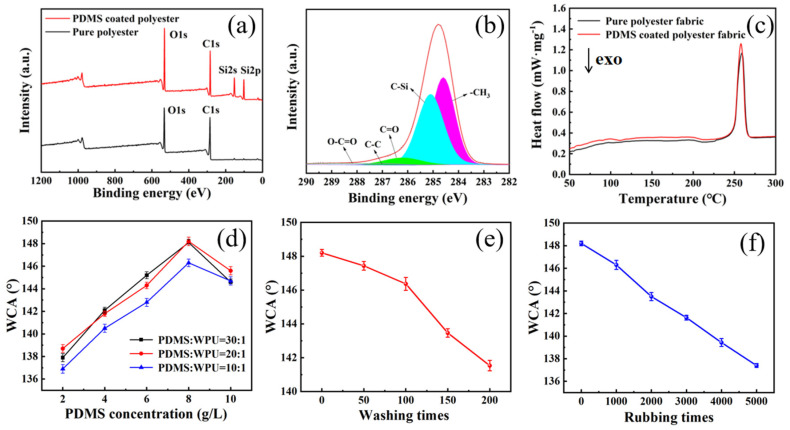
Before and after PDMS coating; (**a**) XPS spectrum, (**b**) C1s peak-splitting narrow spectrum; (**c**) DSC spectrum, the water contact angle of self-cleaning polyester fabric under (**d**) different PDMS content and different mass ratio of PDMS to PU; (**e**) different washing times and (**f**) different rubbing times.

As shown in Figure 3a–e, it can be seen from EDS mapping that the surface of polyester fabric coated with PDMS contains three elements of C, O and Si. However, pure polyester fabric is only composed of C and O elements, and the only source of Si element is PDMS. Therefore, it can be judged that the film covered on the surface of polyester fiber is composed of PDMS. In addition, according to XPS analysis (Figure 4a), pure polyester fabric only contains two elements, C and O, and only the peaks of C 1s and O 1s appear. However, the polyester fabric coated with PDMS contains not only the peaks of C 1s and O 1s but also two new peaks, namely, the peak of Si 2s at 153 eV and the peak of Si 2p at 101 eV. The peak of C 1s is fitted separately (Figure 4b). The results show that the peaks of -CH3, C-Si, C=O, C-C, and O-C=O appear at 284.6, 285.1, 286.3, 286.9, and 288.2 eV, respectively. These chemical bonds are contained in the PDMS and polyester macromolecules, further proving that the polyester fiber is coated by PDMS. As shown in Figure 4c, the melting point of the PDMS coated polyester fabric is slightly lower than that of the pure polyester fabric. This phenomenon can be attributed to the numerous tiny grooves on the surface of the polyester fiber after plasma etching, and PDMS enters these pits, changing its melting point compared with that of the original polyester fiber.

As shown in Figure 4d, when the concentration of PDMS is 8 g/L, the WCA of PDMS-coated polyester fabric is the highest. The reason is that when the concentration of PDMS is lower than 8 g/L, the total amount of low surface energy monomers in the emulsion is too small. During the dipping process, PDMS attached to the surface of polyester fiber is relatively small, resulting in incomplete PDMS film. When the concentration of PDMS is more than 8 g/L, too much PDMS adheres to the surface of polyester fiber during the impregnation process, and the depth of curing-induced crosslinking is limited, and some PDMS cannot participate in the reaction, resulting in insufficient crosslinking. When the mass ratio of PDMS to WPU is 20:1 or 30:1, the hydrophobic effect of PDMS coated polyester fabric is better than that of 10:1. This is because WPU contains certain hydrophilic groups, which leads to the decline of hydrophobic effect. In order to ensure the stability of PDMS emulsion, we prefer 20:1 as the best ratio.

An 8 g/L concentration of PDMS and the mass ratio of PDMS to WPU of 20:1 were selected as the optimal parameter to prepare PDMS coated polyester fabric, the WCA of which reaches 148.2°. The obtained PDMS-coated polyester fabric was washed with different layers (Figure 4e). With the increase of washing times, the WCA gradually decreased, and when the washing times reached 200 times, the WCA decreased to 141.5°. As shown in Figure 4f, the friction resistance of the PDMS coated polyester fabric was tested, and it was found that the WCA gradually decreased with the increase of rubbing times, and the WCA decreased to 138.6° after 5000 rubbing times. The reason for the continuous decline of WCA is that, during the washing or rubbing process of PDMS-coated polyester fabric, firstly, the pleated structure of PDMS film is gradually destroyed, and the surface roughness begins to decrease, and then some of the coating with weak adhesion to the substrate begins to fall off, resulting in defects. Despite 200 times of washing and 5000 times of rubbing, PDMS-coated polyester fabric still maintains good water repellency, which proves that PDMS film is firmly combined with the polyester matrix after atmospheric plasma etching, and has good durability.

**Figure 5:**
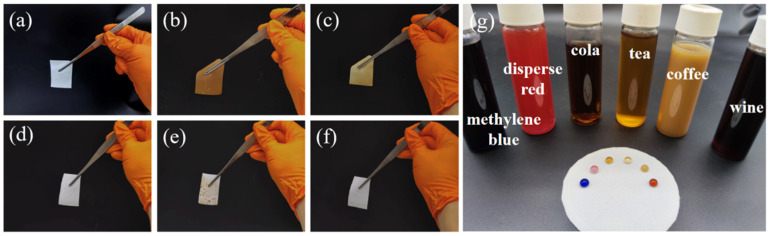
Polyester fabric as it is (**a**) not dipped, (**b**) dipped in soy sauce, (**c**) dipped in deionized water; PDMS coated polyester fabric (**d**) not dipped, (**e**) dipped in soy sauce, (**f**) dipped in deionized water; (**g**) anti-fouling of PDMS coated polyester fabric.

The prepared PDMS coated polyester fabric not only has hydrophobic properties but also self-cleaning and anti-staining functions. Figure 5a–f show that the polyester fabric was soaked in soy sauce. After taking the fabric out, it was completely soaked in soy sauce. After soaking the fabric in deionized water, it remained pale yellow. The PDMS coated polyester fabric was tested according to the same operation. When dipped in soy sauce, the PDMS coated polyester fabric was not wetted by soy sauce and immediately returned to its original white color after dipping in deionized water because the micro-nano fold structure on the surface of PDMS coated polyester fabric can form an air layer between the dirty liquid and the substrate, helping to prevent the fabric from being easily infiltrated by the dirty liquid and achieving an anti-staining performance. Methylene blue dye solution, disperse red dye solution, cola, tea, coffee, and red wine cannot wet the PDMS coated polyester fabric, and all these dirty solutions can maintain a spherical shape on its surface.

**Figure 6:**
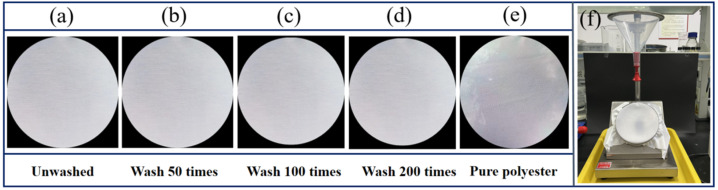
Spray-wetting grade test (**a**) PDMS coated polyester fabric, (**b**) washed 50 times, (**c**) washed 100 times, (**d**) washed 200 times, (**e**) polyester fabric as it is, (**f**) testing device for wetting grade of water.

The wetting grade of the polyester fabric before and after PDMS coating was tested. Figure 6 and Table 1 show that the surface of the original polyester fabric was wetted in a large area, and the wetting grade was only grade 2. However, the polyester fabric coated with PDMS displayed excellent water-drenching and anti-wetting performance, and the wetting grade reached grade 5. After washing 50 times, the wetting remained at grade 5. When the washing increased to 100 times, the wetting grade dropped to grade 4, and the wetting remained at grade 4 after 200 times of washing because the rolling angle of the polyester fabric finished by the PDMS coating was 13.7°, which became 18.6° after 100 times of washing. During the water spraying test, the sample should be kept at 45° with the horizontal plane, and the water drops will quickly slide down when sprayed on the fabric surface. However, after 100 times of washing, a small number of defects appeared on the fabric surface, resulting in the wetting grade drop to level 4.

**Figure 7:**
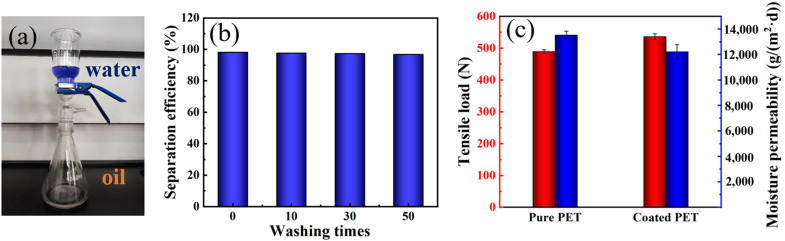
(**a**) Oil–water separation device, (**b**) Oil–water separation efficiency of PDMS coated polyester fabric, (**c**) Tensile load and water vapor transmission of polyester fabric before and after PDMS coating.

As the PDMS surface is hydrophobic and lipophilic, the oil–water separation test was performed on the prepared PDMS coated polyester fabric using the device shown in Figure 7a. Figure 7b shows that the oil–water separation efficiency of the PDMS coated polyester fabric reached 98.37%, and the separation efficiency decreased to 94.67% after 50 times of washing. As shown in Figure 7c, after PDMS coating, the tensile strength of the polyester fabric increased from 489.4 N to 536.4 N compared with the original polyester fabric because a film attached to its surface. After hydrophobic finishing, the water vapor transmission of the polyester fabric decreased slightly from 13,535.7 g/(m^2^·d) to 12,224.3 g/(m^2^·d) because, after coating the polyester fabric with PDMS, some pores were blocked, but it could still conduct moisture transfer through the large number of micropores between the fibers, and the WPU component in PDMS film contained hydrophilic groups, such as -OH, which can realize molecular moisture transfer.

## 4. Conclusions

A durable PDMS hydrophobic coating was constructed on the surface of polyester fiber by a simple process of plasma etching at atmospheric pressure, dipping PDMS emulsion and inducing PDMS crosslinking at high temperature.

(a) The WCA of the self-cleaning polyester fabric is 148.2°, and the wetting grade is 5.
(b) After 200 times of washing, the WCA drops to 141.5°, the wetting grade drops to 4, and the WCA drops to 138.6° after 5000 times of rubbing.
(c) Compared with before and after PDMS coating, the tensile strength of polyester fabric increased from 489.4N to 536.4N, and the water vapor permeability decreased from 13,535.7 g/(m^2^·d) to 12,224.3 g/(m^2^·d).
(d) In addition, we also tested the oil–water separation. The oil–water separation efficiency of the self-cleaning polyester fabric obtained by this method reached 98.37%, and decreased to 94.67% after 50 times of washing.

In the future, on the basis of this research, we will add functional powder to endow self-cleaning polyester fabric with higher hydrophobicity and other properties.

## Figures and Tables

**Table 1:** Test of Wet Grade of Different Polyester Samples.

Samples		Wet Grade
PDMS coated polyester fabric	Unwashed	5
	Wash 50 times	5
	Wash 100 times	4
	Wash 200 times	4
Pure polyester		2

## Data Availability

The data that support the findings of this study are available from the corresponding author upon reasonable request.
